# Predictive value of serum initial brain natriuretic peptide and troponin on functional prognosis in noncardiogenic patients with anterior and posterior circulation cerebral infarction

**DOI:** 10.1055/s-0042-1755270

**Published:** 2022-12-19

**Authors:** Wan-Ting Lu, Wen-Ting Du, De-Sheng Lu, Jie You, Hong-yan Li

**Affiliations:** 1 People's Hospital of Xinjiang Uygur Autonomous Region, Department of Neurology, Urumqi, China.; 2 Xinjiang Medical University, Department of Postgraduate, Urumqi, China.; 3 Shihezi People's Hospital, Department of Neurosurgery, Shihezi, China.

**Keywords:** Infarction, Anterior Cerebral Artery, Brain Infarction, Natriuretic Peptide, Brain, Troponin, C-Reactive Protein, Prognosis, Embolism, Infarto da Artéria Cerebral Anterior, Infarto Encefálico, Peptídeo Natriurético Encefálico, Troponina, Proteína C-Reativa, Prognóstico, Embolia

## Abstract

**Background** Brain natriuretic peptide (BNP) and troponin have a close relationship with cardiogenic cerebral embolism (CCE), but their relationship with noncardiogenic patients with anterior circulation ischemia (ACI) and posterior circulation ischemia (PCI) is not clear.

**Objective** To explore the predictive value of serum initial BNP and troponin on the functional prognosis of patients with noncardiogenic ACI and PCI.

**Methods** Consecutive patients with first-episode cerebral infarction within 12 hours of symptom onset were enrolled in the present 1-year prospective cohort study. Serum levels of BNP and troponin were collected within 12 hours of onset. Infarction location was classified as ACI and PCI by magnetic resonance imaging (MRI). According to the modified Rankin Scale (mRS) score at 90 days after onset, ACI and PCI cases were respectively divided into a good prognosis group (mRS score between 0 and 2) and a poor prognosis group (mRS score between 3 and 6). The general state of health and results of laboratory examinations and other auxiliary examinations of all patients were recorded. Single-factor analysis and multivariate logistic regression analysis were used to assess the relationship between serum levels of BNP, troponin, and functional outcome.

**Results** The multivariate logistic regression found that higher levels of initial BNP (odds ratio [OR] = 1.024; 95% confidence interval [CI]: 1.006–1.041; *p* = 0.007) and C-reactive protein (CRP) (OR = 1.184; 95%CI: 1.024–1.369; *p* = 0.022) were independent predictors of poor functional prognosis of noncardiogenic PCI at 90 days after onset after adjusting for age, gender, ethnicity, history of hypertension and of diabetes.

**Conclusions** The levels of initial BNP and CRP were related to poor functional outcomes in noncardiogenic PCI patients at 3 months, independent of troponin.

## Resumo

**Antecedentes** O peptídeo natriurético cerebral (BNP, na sigla em inglês) e a troponina estão intimamente relacionados com a embolia cerebral cardiogênica (CCE, na sigla em inglês), mas a relação com pacientes não cardioembólicos com isquemia de circulação anterior (ICA) e isquemia de circulação posterior (ICP) não é clara.

**Objetivo** Investigar o valor preditivo dos níveis séricos iniciais do BNP e da troponina no prognóstico de pacientes com AVC isquêmico não cardiogênico.

**Métodos** Os níveis séricos de BNP e de troponina foram recolhidos de pacientes com primeiro episódio de acidente vascular cerebral (AVC) isquêmico dentro de 12 horas após o início dos sintomas, com localização classificada como ICA e ICP de acordo com exame de ressonância magnética (RM). De acordo com a pontuação modificada da escala de Rankin (mRS), aos 90 dias após o início dos sintomas, ICA e ICP foram divididas respectivamente em um grupo de bom prognóstico (mRS entre 0 e2) e em um grupo de mau prognóstico (mRS entre 3 e 6). Foram registrados exames laboratoriais e outros exames complementares de todos os pacientes. Foram utilizadas análise fatorial única e análise de regressão logística multivariada para investigar a relação entre os níveis séricos de BNP e de troponina e o resultado funcional.

**Resultados** A regressão logística multivariada evidenciou que os níveis mais altos de BNP inicial (odds ratio [OR] = 1,024, intervalo de confiança [IC] de 95%: 1,006–1,041; *p* = 0,007) e proteína C reativa (CRP, na sigla em inglês) (OR = 1,184; 95%CI: 1,024–1,369; *p* = 0,022) foram preditores independentes de mau prognóstico funcional da ICP não cardiogênica aos 90 dias após o início dos sintomas.

**Conclusões** Os níveis iniciais de BNP e CRP se associaram a maus resultados funcionais em pacientes com ICP não cardiogênica aos três meses, independentemente da troponina.

## INTRODUCTION

Stroke is one of the most common causes of death and disability in China. 1 It is estimated that the incidence of cerebrovascular events in China will increase by ∼ 50% in 2030 compared with 2010. 2 Acute ischemic strokes (AISs) account for ∼ 70% of all strokes, 3 and their morbidity, recurrence rate, mortality, and economic burden are far beyond those of hemorrhagic stroke. 4

According to the clinical symptoms and neuroimaging, anterior circulation ischemia (ACI) indicates middle cerebral artery and anterior cerebral artery system ischemia, 5 while posterior circulation ischemia (PCI) is defined as ischemic infarction of the area of the vertebrobasilar arterial system that supplies the posterior half of the cerebral hemisphere, the interbrain, the brainstem (48%), and the cerebellum. 6 Due to the different vascular anatomy and easy embolization location of the posterior and anterior circulatory systems, their clinical characteristics, etiology, and prognostic factors are also diverse.

At present, data comparing the prognosis of PCI and ACI are limited. Posterior circulation ischemia only accounts for between 20 and 25% of all ischemic strokes, and the National Institutes of Health Stroke Scale (NIHSS) is far less accurate in assessing the severity of PCI than that of ACI, which may lead clinicians to pay less attention to PCI. 7 Although the treatment for AIS has improved, the mortality and disability rate of PCI is still close to 80%. 8 Thus, more sensitive PCI biomarkers are urgently needed to predict and improve the prognosis of PCI patients as early as possible.

In the Hallym stroke registry, only 11% of the 591 Korean patients with PCI were led by cardiogenic embolism. 9 In another study of 2,545 Chinese patients, cardiogenic cerebral infarction accounted for 5.4% of PCI patients, which is significantly lower than ACI, at 13.3%. 10 These studies showed that noncardiogenic embolization might be the leading cause of posterior circulation cerebral infarction, which is different from the main cause of anterior circulation cerebral infarction. Therefore, further studies are needed.

As is well known, brain natriuretic peptide (BNP) and troponin (cTn-T; cTn-I) are favorable serum markers for detecting myocardial injury. Because of their close relationship with cardiogenic cerebral embolism (CCE), neurologists are paying increasing attention to them. 11 James et al. 12 first discovered that troponin could not only assess the severity of acute stroke patients but also indicate the prognosis of the disease. Maȧkikallio et al. 13 found that elevated serum BNP was an independent risk factor for death in patients with cerebral infarction. However, the relationship among noncardiogenic stroke, BNP, and cTn is controversial. Gupta et al. 14 and Barber et al. 15 showed that serum BNP and cTn levels were unrelated to the functional prognosis of noncardiogenic cerebral infarction. Zhao et al. 16 and Nigro et al. 17 believed that BNP and cTn were reliable prognostic markers of noncardiogenic cerebral infarction. Remarkably, lesion location differences were not considered in the aforementioned studies. Therefore, the present paper aimed to explore whether plasma BNP and troponin can be used as simple, convenient, specific and economical serum markers to predict the functional prognosis in noncardiogenic patients with ACI and PCI.

## METHODS

### Patients

The present study was a 1-year prospective study. From May 2018 to June 2019, data from consecutive patients with acute cerebral infarction 18 confirmed by computed tomography (CT) or magnetic resonance imaging (MRI) (DWI) admitted to the Department of Neurology and to the Emergency Department of the People's Hospital of Xinjiang Uygur Autonomous Region, China, were collected and recorded. The patients were first-time stroke patients, ≥18 years old, and the time of symptom onset was ≤ 12 hours. 15 The exclusion criteria were: (1) thrombolysis or interventional therapy; (2) potential or existing heart disease: abnormalities with examination by standard 12-lead electrocardiogram (ECG), stroke monitoring ward ECG monitoring, 24-hour dynamic electrocardiogram, chest X-ray, echocardiography and transesophageal echocardiography at admission; (3) infarction combined with cerebral hemorrhage; (4) simultaneous ACI and PCI; (5) head CT or MRI examination revealing intracranial hemorrhage, subarachnoid hemorrhage, subdural hematoma, or nervous system tumor-induced ischemic stroke; (6) undetermined stroke subtype; (7) modified Rankin Scale (mRS) score ≥ 3 points before onset combined with serious heart or lung disease, malignant tumors and other serious infections, renal dysfunction, seizures, pregnancy or pulmonary heart disease; and (8) inability to cooperate with follow-up. Informed consent was obtained from all enrolled patients.

### Clinical information collection

Clinical information was collected for all enrolled patients, including demographic data, cerebral infarction risk factors, current incidence, cranial imaging, and laboratory results. The NIHSS was used to assess clinical severity at admission. The neurological function scores of all patients at admission were evaluated by two neurologists at the associate senior level or above for selected patients with AIS.

The relevant staff of the Stroke Screening Base of the Department of Neurology conducted a telephone follow-up or face-to-face follow-up of the outpatient clinic 3 months after the onset of the disease. The mRS score was recorded as mRS 90d, and 4 cases were lost to follow-up. All patients underwent DWI, MRI, CTA, and carotid ultrasonography. The infarct was classified based on its location as ACI and PCI via cranial imaging. Based on the mRS 90d score obtained at the follow-up, the 2 groups of patients were respectively divided into a good prognosis group (mRS score between 0 and 2) and a poor prognosis group (mRS score between 3 and 6).

### Laboratory testing

Fingertip blood glucose (Boshi blood glucose meter, TD4279A) was measured in all enrolled patients within 12 hours after admission. Serum BNP concentration was rapidly measured by Boshi Triage bedside rapid quantitative myocardial infarction and heart failure diagnostic instrument (Biosite Systems Ltd., Solihull, UK). Troponin was detected by double antibody sandwich enzyme-linked immunosorbent assay (ELISA), and the kit was provided by Fujian Maixin biological technology co. LTD. D-dimer was detected by immunoturbidimetry with automatic hemagglutination analyzer (cissm CA7000, Japan). Bilirubin, aminotransferase, and creatinine were determined by an automatic dry chemical analyzer (vitros-5600, Johnson & Johnson, New Brunswick, NJ, USA). The reference values of natriuretic peptides and cardiac troponins assays were: BNP: 0 to 100 pg/ml; cTnT: 0 to 0.014 ng/ml); cTnI: 0 to 0.034ng/ml.

### Statistical analysis

IBM SPSS Statistics for Windows, version 23.0 (IBM Corp., Armonk, NY, USA) was used for statistical analysis. A normality test was conducted for the measured data. The data conforming to the normal distribution were expressed as means ± standard deviations (SDs), including age, body mass index (BMI), etc. The composition ratio and rate were used to describe the basic characteristics of the enumeration data, such as gender, ethnicity, history of hypertension, etc. Two independent sample *t* -tests were used to compare the means between the two groups. Non-normal distribution data after data conversion were represented by medians (25%, 75%), and the Wilcoxon nonparametric test was used for intergroup comparison. After univariate analysis of baseline data, vascular disease risk factors, stroke subtypes, and possible risk factors in the group with good prognosis of ACI and PCI (mRS between 0 and 2) and the group with poor prognosis (mRS between 3 and 6), variables with a p-value < 0.1 were incorporated into the multivariate logistic regression model for analysis. The relative risk of the ACI and PCI groups was expressed by odds ratio (OR) (95% confidence interval [CI]). Both groups were tested. GraphPad Prism 8 (GraphPad Software, San Diego CA, USA) was used to plot the receiver operating characteristic (ROC) curve. After ROC curve analysis, the optimal cutoff values of each variable were obtained. In the present study, *p* < 0.05 was considered statistically significant.

## RESULTS

During the inclusion period, 1,424 ACS patients were screened. A total of 79 PCI patients and 69 ACI patients were included. Out of the total, 320 patients with onset of symptoms > 12 hours, 517 with thrombolysis or mechanical thrombectomy treatment, 3 without informed consent, 143 with cardiogenic stroke, 19 78 with severe inflammation, 23 with kidney failure, 2 with brain lupus, 52 with hemorrhagic infarction, 27 with undetermined stroke subtype or with rare causes of stroke subtype, such as Moya Moya disease, and 110 with PCI merged with ACI infarction were excluded. A total of 148 patients completed the follow-up (4 were lost to follow-up). All enrolled patients were treated with standardized anticoagulant and antiplatelet therapy after discharge.

### Comparison of baseline characteristics between good and poor prognosis groups with anterior and posterior circulation ischemia

The general information of ACI patients is provided in ( Table 1 ), and that of PCI patients is provided in ( Table 2 ). For ACI patients, the 2 groups of patients were similar in terms of baseline data(age [ *p* = 0.201], gender [ *p* = 0.303], Glasgow Coma Scale (GCS) at admission [ *p* = 0.192]. In PCI patients, age ( *p* = 0.113), gender ( *p* = 0.132), and GCS at admission ( *p* = 0.192) were similar.

**Table 1:** Clinical data of anterior circulation ischemia according to prognosis

	Total	Good prognosis	Poor prognosis	p-value
*n*	69	47	22	−
Age, years od x̅ ± SD		60.24 ± 13.92	67.14 ± 14.02	0.201
Sex, *n* (%)		(23/24)	(7/15)	0.303
BMI, kg/m2, x̅ ± SD		26.72 ± 3.19	25.88 ± 3.72	0.577
Smoking, *n* (%)		24 (51.6)	14 (63.6)	0.901
Drinking *,* *n* (%)		7 (2.1)	1 (4.5)	0.712
Prior vascular risk factors, *n* (%)				
Hypertension		30 (63.8)	11 (50.0)	0.672
Diabetes mellitus		27 (57.4)	10 (79.1)	0.702
Hypercholesterolemia		39 (70.9)	15 (45.4)	0.603
NIHSS at admission, median (IQR)		6.00 (1.00–15.00)	13.50 (7.00, 15.00)	0.031
GCS at admission, x̅ ± SD		7.72 ± 6.231	10.92 ± 6.723	0.192
Stroke subtype				0.238
Large artery atherosclerosis	37	27 (57.4)	10 (45.4)	
Small vessel occlusion	32	25 (53.1)	7 (31.8)	
Laboratory findings				
BNP, pg/nl		25.4 (13.45–77.26)	32.20 (13.10–149.80)	0.353
cTnI, ng/L		0.01 (0.003–0.012)	0.004 (0.003–0.012)	0.319
cTnT, ng/L		0.012 (0.007–0.022)	0.011 (0.007–0.022)	0.791
IL-6, mmol/L		4.40 (2.855–6.895)	6.63 (4.53–11.29)	0.031
Glucose level, mmol/L		6.53 (5.715–9.41)	7.670 (6.00–9.23)	0.704
BUN, mg/L		5.50 (4.20–7.05)	5.62 (4.90–7.20)	0.425
Cr, mg/L		63.90 (51.40–76.10)	63.80 (56.20–74.60)	0.872
TBIL, μmol/L		12.50 (7.59–16.50)	13.285 (10.36–19.00)	0.135
ALT, U/L		18.00 (11.50–28.00)	18.50 (14.00–30.00)	0.602
GOT, U/L		16.00 (13.00–25.00)	18.50 (14.00–27.00)	0.523
D-dimer, mg/l		0.30 (0.23–0.525)	0.53 (0.29–1.27)	0.053
CRP, mg/l		3.51 (2.50–6.66)	6.365 (2.51–13.80)	0.041

Abbreviations: ALT, alanine transaminase; BNP, brain natriuretic peptide; BUN, blood urea nitrogen; Cr, creatinine; CRP, C-reactive protein; cTnI, troponin I; cTnT, troponin T; GCS, Glasgow Coma Scale; GOT, glutamic oxalacetic transaminase; IL-6: interleukin-6; NIHSS, National Institutes of Health Stroke Scale; SD, standard deviation; TBIL, total bilirubin.

**Table 2:** Clinical data of posterior circulating ischemia according to prognosis

	Total	Good prognosis	Poor prognosis	p-value
n	79	55	24	−
Age, years old x̅ ± SD		59.44 ± 14.977	65.21 ± 13.609	0.113
Sex, *n* (%)		(30/25)	(9/15)	0.132
BMI, kg/m2, x̅ ± SD		25.77 ± 4.23	24.92 ± 3.69	0.242
Smoking, *n* (%)		13 (23.6)	6 (25)	0.721
Drinking, *n* (%)		5 (9.0)	1 (4.0)	0.820
Prior vascular risk factors, *n* (%)				
Hypertension		37 (67.2)	20 (83.3)	0.954
Diabetes mellitus		18 (32.7)	19 (79.1)	0.702
Hypercholesterolemia		39 (70.9)	15 (62.5)	0.532
NIHSS at admission, median (IQR)		3.00 (1.00–15.00)	9.50 (2.50–14.50)	0.456
GCS at admission, x̅ ± SD		9.91 ± 6.631	11.83 ± 5.623	0.192
Stroke subtype				0.102
Large artery atherosclerosis	49	38 (69.0)	11 (45.8)	
Small vessel occlusion	30	22 (40.0)	8 (33.3)	
Laboratory findings				
BNP, pg/nl		27.20 (10.50–71.90)	134.60 (60.65–190.21)	0.000
cTnI, ng/L		0.005 (0.002–0.012)	0.011 (0.005–0.013)	0.034
cTnT, ng/L		0.009 (0.007–0.014)	0.015 (0.009–0.019)	0.013
IL-6, mmol/L		3.75 (2.31–5.95)	9.82 (7.78–24.50)	0.000
Glucose level, mmol/L		6.36 (5.62–8.11)	7.15 (6.00–9.65)	0.200
BUN, mg/L		5.30 (4.43–6.05)	6.15 (4.20–8.10)	0.166
Cr, mg/L		63.50 (54.75–69.95)	62.30 (5'0.80–84.75)	0.721
TBIL, μmol/L		13.37 (10.24–19.01)	13.98 (7.97–19.85)	0.737
ALT, U/L		23.00 (17.00–31.00)	15.00 (8.50–20.00)	0.001
GOT, U/L		20.00 (15.50–24.00)	16.50 (13.99–21.00)	0.039
D-dimer, mg/l		0.31 (0.21–0.47)	0.720 (0.39–1.22)	0.000
CRP, mg/l		2.50 (2.41–4.15)	5.30 (3.00–12.53)	0.002

Abbreviations: ALT, alanine transaminase; BNP, brain natriuretic peptide; BUN, blood urea nitrogen; Cr, creatinine; CRP, C-reactive protein; cTnI, troponin I; cTnT, troponin T; GCS, Glasgow Coma Scale; GOT, glutamic oxalacetic transaminase; IL-6, interleukin-6; NIHSS, National Institutes of Health Stroke Scale; SD, standard deviation; TBIL, total bilirubin.

A correlation between the NIHSS score, interleukin-6 (IL-6), CRP at admission, and poor prognosis of ACI patients was found ( *p* < 0.05). However, after multifactor analysis ( Table 3 ), the NIHSS score (OR = 1.095; 95%CI: 0.999–1.201; *p* = 0.054), IL-6 (OR = 0.974; 95%CI: 0.897–1.057; *p* = 0.527), and CRP (OR = 0.057; 95%CI: 0.977–1.147; *p* = 0.167) were not independent risk factors for poor prognosis of ACI.

**Table 3:** Multivariate analyses of risk factors affecting the prognosis of anterior circulation ischemia

	OR	95%CI	p-value
NIHSS at admission	1.095	0.999–1.201	0.054
IL-6, mmol/L	0.974	0.897–1.057	0.527
CRP, mg/l	0.057	0.977–1.147	0.164

Abbreviations: CI, confidence interval; CRP, C-reactive protein; IL-6, interleukin-6; NIHSS, National Institutes of Health Stroke Scale.

In the PCI groups, unlike the ACI groups, the NIHSS score ( *p* = 0.456) was unrelated to the prognosis of the PCI group. The BNP, cTnI, cTnT, IL-6, ALT, GOT, D-Dimer, and CRP levels were all related to PCI poor outcome ( *p* < 0.05). After multifactor analysis ( Table 4 ), the data suggested negative correlations between poor prognosis and initial BNP (OR = 1.024; 95%CI: 1.006–1.041; *p* = 0.007) and CRP (OR = 1.184; 95%CI: 1.024–1.369; *p* = 0.022).

**Table 4:** Multivariate analyses of risk factors affecting the prognosis of posterior circulation ischemia

	OR	95%CI	p-value
BNP, pg/nl	1.024	1.006–1.041	0.007
cTnI, ng/L	1.986	0–4.293	0.452
cTnT, ng/L	0.000	0–3.918	0.639
IL-6, mmol/L	1.089	0.98–1.21	0.113
ALT, U/L	0.961	0.837–1.104	0.574
GOT, U/L	0.981	0.785–1.226	0.864
D-Dimer, mg/l	1.197	0.546–2.624	0.654
CRP, mg/l	1.184	1.024–1.369	0.022

Abbreviations: ALT, alanine transaminase; BNP, brain natriuretic peptide; CI, confidence interval; CRP, C-reactive protein; cTnI, troponin I; cTnT, troponin T; GOT, glutamic oxalacetic transaminase; IL-6: interleukin-6.

### Association between initial BNP and cTn levels and prognosis of anterior and posterior circulation ischemia

**Figure 1:**
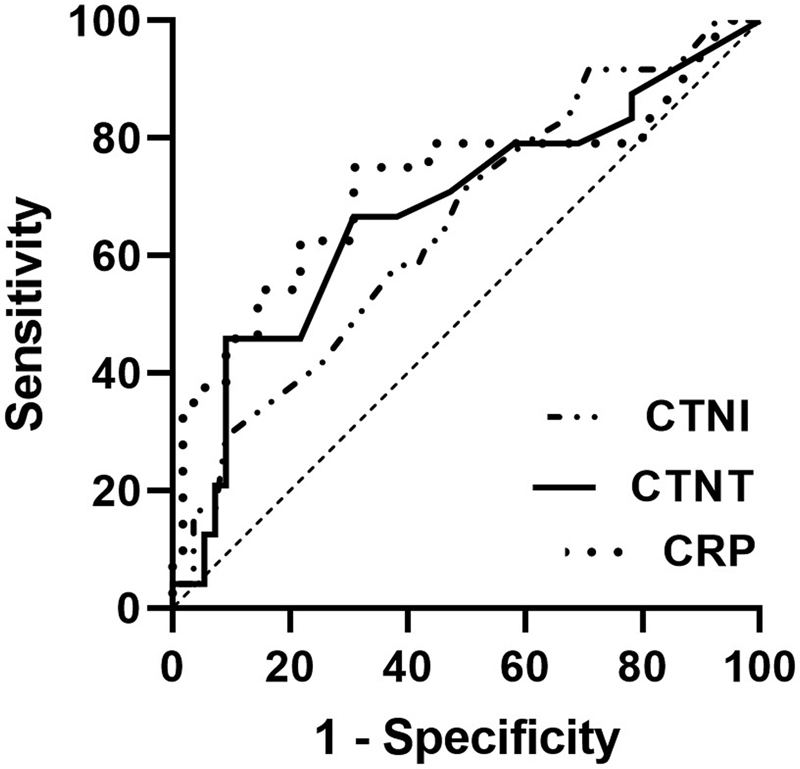
ROC curve between cTn, CRP and PCI patients' poor prognosis. Abbreviations: cTnI, Troponin I; cTnT, Troponin T; CRP, C-reactive Protein.

**Figure 2:**
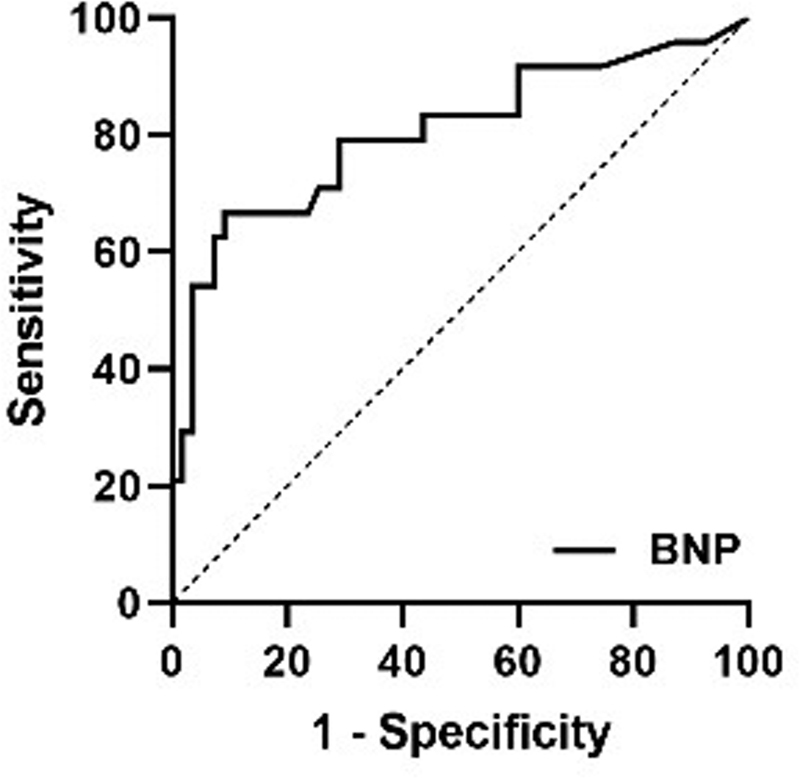
ROC curve between BNP and PCI patients' poor prognosis. Abbreviation: BNP, Brain Natriuretic Peptide.

The present study showed that initial cTn levels could not determine the prognosis of either ACI or PCI patients at 3 months. Among the 69 noncardiogenic ACI patients, initial BNP levels had no relation with a 90-day poor prognosis in ACI patients ( *p >* 0.05). Among 79 noncardiogenic PCI patients, initial BNP and cTn were related with a poor prognosis for PCI ( *p* < 0.05), with a ROC curve ( Figure 1 ) with the area under the curve (AUC) of 0.650 for cTnI (95%:CI: 0.519–0.781) and of 0.675 for cTnT (95%:CI: 0.538–0.812). However, only initial BNP (OR = 1.024; 95%CI: 1.006–1.041; *p* = 0.007) was an independent risk factor for a poor prognosis in noncardiogenic PCI patients. With a ROC curve ( Figure 2 ) with an AUC of 0.803 (95%CI: 0.684–0.922), BNP showed a better predictive ability for poor outcomes in noncardiogenic PCI patients (cutoff value: 94.96 pg/nl; sensitivity: 66.7%; specificity: 90.9%; *p* = 0.000).

## DISCUSSION

Previous studies showed that serum BNP 3 20 and troponin 21 in patients with AIS were related to the occurrence and development of AIS and were more reliable biomarkers for the diagnosis of cardioembolic cerebral infarction and for the evaluation of its prognosis. 22 In recent years, studies found that BNP and cTn could also predict the short-term prognosis of patients with noncardiogenic cerebral infarction. 23 The increase of BNP and cTn levels in patients with noncardiogenic cerebral infarction might not be accompanied by an increase in ventricular volume load or abnormalities in ventricular function, but they were related to autonomic nervous disorder, imbalance of sympathetic vagus nerve regulation, secretion of endogenous vasoconstrictor and inflammatory mediators, hypothalamic ischaemia, 24 hypoxemia, and other factors. 25 A large number of studies 26 27 found that after autonomic nervous disorder, sympathetic and parasympathetic nervous regulation were out of balance, which affected the hypothalamic-pituitary-adrenalin axis and changed the level of catecholamine, stimulating the production of BNP and cTn. Bieber et al. 28 showed that, after acute infarction in the right cerebral hemisphere, increased sympathetic nerve activity resulted in increased blood catecholamine and cortisol levels and faster heart rate, which could induce chronic myocardial systolic dysfunction, as well as increased blood BNP and troponin levels. 29

Ay et al. 30 showed that the incidence of increased cTn (cTnI; cTnT) in the insular infarction group was higher than that in the noninsular infarction group. The incidence of increased cTn in patients with right insular infarction was higher than in those with left insular infarction. The right insula was the sympathetic control area, and the left insula was the parasympathetic control area. When excessive sympathetic stimulation interfered with cardiac autonomic nervous activity, rapid arrhythmia occurred frequently, which might lead to brain-derived myocardial injury, making the prognosis of patients with right insular infarction worse. At the same time, the present study also suggested that except for the right insula, which is the highest center of autonomic nerves, the hypothalamus, the brain stem, and other brain regions with active autonomic nerves might also be related to the increase of cTnT, which also became an opportunity for our study. If autonomic nerve dysfunction causes serum cTn levels to rise, is the increase of cTn more obvious when an autonomic nerve rich area (posterior circulatory system) is injured and is the correlation with prognosis stronger? Our study found that troponin was not an independent risk factor for poor prognosis in patients with noncardiogenic PCI, but it was associated with poor prognosis in posterior circulation, which was consistent with the study by Hasırcı et al. 31 For patients with PCI, the sympathetic nerve was overactive, resulting in the imbalance of sympathetic and vagal nerve regulation, which might lead to a poor prognosis. However, troponin had no correlation with ACI, which was consistent with the study by Abdi et al. 32

Fang et al. 33 found that the plasma BNP level was significantly higher in the PCI group than in the ACI group. The initial plasma BNP level was of a certain reference value for the prognosis of PCI, 13 24 which was consistent with our findings. However, Menon et al. 34 believed that plasma BNP could predict the prognosis of 90 days of total ACI and had no special significance in the posterior circulation group. Due to the small sample size of each group in this study, there are differences from our study. Our study showed that plasma BNP level of 94.96 pg/ml at admission could be used as a predictor of poor prognosis in the posterior circulation group, with a sensitivity of 66.70% and specificity of 90.90%. Studies have shown that the brain tissues in the posterior circulation area that are rich in BNP (such as the medulla oblongata and the hypothalamus) can also raise plasma BNP and cTn when damaged, in addition to the autonomic nervous system disorder. When the aforementioned parts are injured, the neurons in the brain tissue around the infarct will be stimulated to secrete BNP, causing a large amount of neurogenic BNP to enter the blood by the damaged blood-brain barrier. Meanwhile, combined with the multivariate logistic regression analysis of the prognosis of patients with PCI at 90 days, the study showed that initial BNP was significantly correlated with a poor prognosis in patients with PCI ( *p* < 0.05), but this correlation was not found in the ACI group. Therefore, we can speculate that the release of neurogenic BNP into the blood is related to the poor prognosis of noncardiogenic PCI stroke. Determination of the initial BNP concentration can predict the prognosis of noncardiogenic PCI stroke to a certain extent.

Autonomic dysfunction after AIS is a potential cause of impaired physiological regulation of heart rate and blood pressure, as well as of increased secretion of catecholamine and cortisol. Increased sympathetic activity is associated with poor prognosis, and a slight increase in plasma BNP and cTn may be a more intuitive indication of autonomic dysfunction and potential myocardial damage. Reducing sympathetic nerve or enhancing the activity of the vagus nerve may be an effective therapeutic approach. Studies 28 demonstrated that the antisympathetic treatment of the β-2 antagonist metoprolol could prevent autonomic disorders and the development of chronic cardiac dysfunction. Plasma BNP levels also decreased with the use of β-blockers, suggesting that autonomic nervous function may be regulated by lowering serum BNP and cTn levels and that poor prognosis may be improved. At present, there is no good treatment method for PCI. Therefore, it may be of great significance to explore the changes in the blood concentration of BNP after acute PCI and whether BNP released by ischemic brain tissue can play a neuroprotective role around ischemic areas.

In addition, our study also found that, unlike the patients with acute noncardiogenic precirculatory infarction, the elevation of initial serum CRP also has a certain evaluation value for the prognosis of patients with acute noncardiogenic precirculatory infarction. The relationship between acute cerebral ischemia and inflammatory response activation is complex. C-reactive protein-activated leukocyte inflammation factors, such as the synthesis of endothelial cells to promote damaged brain tissue inflammation, is aggravating; by increasing the level of platelet activating factor, and inhibition of former dissolving enzyme original activator to promote thrombosis and unstable plaque formation, damage further aggravates atherosclerosis, producing a variety of thrombosis complications. 35 The latest research shows that high serum CRP levels on admission to hospital predict poor prognosis of acute cerebral infarction; elevated CRP is a reliable marker of patients who are more likely to relapse 36 and become emotional after stroke. Cognitive impairment and depression are more likely. 37 At the same time, it was assumed that high CRP levels are independent risk factors for the prognosis of cardiac cerebral infarction. 2 The results of the present study show that the cardiac cycle after cerebral infarction patients are admitted to hospital with higher levels of CRP may predict the function of patients after 90 days of poor prognosis. In the literature, treatment of noncardiac cerebral infarction patients with pravastatin found that pravastatin cannot only improve the prognosis of patients with PCI but also reduce the risk of recurrence. This suggests that statins can not only reduce blood fat but also be generated by reducing serum CRP level anti-inflammatory effects 38 because the PCI and ACI before treatment are the same, but the onset of PCI is more hidden. Nerve function defect symptoms are not typical; the NIHSS score is difficult to use to make an accurate assessment of severity, leading to the inability to give timely treatment monitoring and lower CRP levels, which may help to improve the prognosis of circulation of cerebral infarction after acute noncardiac, and be favorable for individualized treatment decisions.

In conclusion, the present study confirmed that the initial BNP level was temporarily increased in serum of patients with noncardiogenic cerebral infarction unrelated to heart disease, which could be used as a useful biomarker for poor prognosis of reactive noncardiogenic PCI. The previous studies 17 24 revealed that oral anticoagulant therapy could more greatly benefit patients with PCI with increased BNP compared with antiplatelet therapy. The underutilization of β antagonists may explain the increased adverse outcomes observed in patients with acute noncardiogenic PCI, which needs to be further confirmed and guided by further clinical trials. More basic and clinical studies are needed to clarify the possible mechanism of the slight increase in BNP and its potential role in acute noncardiogenic PCI. When serum BNP is elevated in patients, antisympathetic and cardiac therapy should be considered more carefully to prevent and improve the poor functional outcomes of patients.
